# Insight into pericytes in glioblastoma angiogenesis: In vivo tracking by two‐photon microscopy and proteomic profiling

**DOI:** 10.1002/ame2.70073

**Published:** 2025-08-07

**Authors:** Qinghong Wang, Chengyan Ma, Xinpei Wang, Mengyuan Li, Xingjiu Yang, Ran Gao

**Affiliations:** ^1^ Institute of Laboratory Animal Science, CAMS & PUMC, National Human Diseases Animal Model Resource Center, National Center of Technology Innovation for Animal Model Beijing China; ^2^ State Key Laboratory of Respiratory Health and Multimorbidity, CAMS & PUMC Beijing China; ^3^ NHC Key Laboratory of Comparative Medicine, CAMS & PUMC Beijing China

**Keywords:** angiogenesis, glioblastoma, pericytes, tumor microenvironment, two‐photon microscopy

## Abstract

**Background:**

Glioblastoma (GBM) is a highly aggressive brain tumor characterized by aberrant angiogenesis and an immunosuppressive microenvironment. Pericytes are aberrantly recruited but their spatiotemporal roles and molecular changes remain unclear. This study investigated platelet‐derived growth factor receptor beta‐positive (Pdgfrb^+^) pericyte dynamics and reprogramming in GBM vasculature.

**Methods:**

We generated GL261‐Luc and GL261‐CFP glioblastoma cells via lentiviral transduction and established two transgenic models. (1) For pericyte labeling, Ai14 reporter mice was crossed with *PDGFRβ‐P2A‐CreER* ^*T2*^ mice for *tdTomato*‐specific lineage tracing (PT mice). (2) For conditional ablation, we generated inducible *Pdgfrb*‐expressing cell ablation models (PT mice was crossed with *ROSA‐DTA* mice). An intravital imaging platform (FITC‐dextran/CFP/tdTomato + two‐photon microscopy) tracked pericytes, vessels, and tumor cells, while FACS‐sorted Pdgfrb^+^ cells from GBM and normal brain were analyzed by LC–MS/MS proteomics.

**Results:**

Cre‐mediated ablation of *Pdgfrb*‐expressing cells revealed stage‐dependent effects on GBM growth: early ablation inhibited progression while late ablation promoted it. Pericytes undergo dual spatial reorganization in GBM: regional enrichment with pre‐sprouting accumulation at the tumor‐brain interface, and focal positioning with preferential localization at vascular branch points. Concurrently, GBM vasculature displayed simplified branching, dilation, and pericyte remodeling (shorter processes, higher density). Proteomics revealed 1426 altered proteins, with upregulated proliferation pathways (e.g., matrix metallopeptidase 14 [Mmp14], lysyl oxidase like 2 [Loxl2]) and downregulated homeostasis functions (e.g., transforming growth factor beta 1 [Tgfb1]), validated by scRNA‐seq in human GBM.

**Conclusions:**

This study demonstrates that during early GBM progression, pericytes actively drive tumor angiogenesis through molecular reprogramming toward proliferative and pro‐angiogenic phenotypes, with the integrated imaging‐proteomics framework revealing potential therapeutic targets for disrupting pericyte‐mediated vascular remodeling.

## Graphical abstract

Based on the inducible pericyte ablation model, we discovered that Pdgfrb^+^ pericytes regulate glioblastoma (GBM) growth. Using the pericyte lineage tracing model, we revealed their dynamic behavior during GBM angiogenesis. Proteomic analysis combined with pericyte RNA analysis based on human single‐cell data demonstrated cross‐species conserved alterations, suggesting these changes represent promising therapeutic targets for pericyte‐targeted GBM treatment.

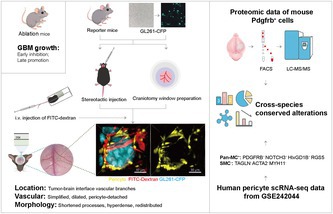

## 1 INTRODUCTION

Glioblastoma (GBM), the most common and aggressive primary brain tumor, accounts for over 50% of all malignant brain tumors.^1^ Despite standard treatments including surgical resection, radiotherapy, and temozolomide chemotherapy, GBM prognosis remains extremely poor, with a five‐year survival rate of only 5.8% and nearly inevitable recurrence.^2^ While immunotherapies show limited efficacy due to the immunosuppressive tumor microenvironment, blood–brain barrier restrictions, and tumor heterogeneity,^3^ future research will focus on multi‐target inhibitors.

A hallmark of GBM progression is pathological angiogenesis,^4^, ^5^, ^6^ though the dynamic remodeling mechanisms remain incompletely understood. Traditional anti‐angiogenic drugs like bevacizumab that target VEGF signaling temporarily reduce tumor perfusion but fail to improve overall survival due to adaptive resistance mechanisms.^7^, ^8^ Tumors evade VEGF inhibition by activating compensatory pathways such as FGF and Angiopoietin‐2, or through vessel co‐option where tumor cells hijack existing vasculature.^9^, ^10^, ^11^, ^12^, ^13^, ^14^

Pericytes, essential vasculature components that encircle blood vessel endothelial linings, are pivotal regulators of vascular integrity.^15^ In healthy brain vasculature, they maintain blood–brain barrier (BBB) stability through direct contact and paracrine signaling.^16^, ^17^ However, in GBM, pericytes undergo pathological reprogramming.^18^, ^19^, ^20^, ^21^, ^22^, ^23^ Preclinical studies demonstrate that dual inhibition of pericytes (via PDGFRβ) and endothelial cells (via VEGFR, vascular endothelial growth factor receptor) enhances anti‐neovascularization effects and reduces tumor invasion.^15^ Targeting pericytes in combination with other therapeutic modalities offers a promising strategy for GBM treatment.

To address these questions, we integrated longitudinal two‐photon imaging with proteomics analysis. Although *PDGFRβ* is expressed in both vascular smooth muscle cells and glial cells, pericyte subpopulations were precisely identified through anatomical localization and morphological characteristics.^15^, ^24^ By combining cranial window surgery with two‐photon microscopy, this study for the first time captures in real‐time the dynamic behavior of GBM‐associated pericytes and their real‐time correspondence with vascular morphological changes in vivo via two‐photon microscopy. This approach overcomes the inherent limitations of static models and provides visual evidence for understanding the role of pericytes in vascular remodeling. Bioinformatics analysis elucidated the molecular signatures of tumor‐associated pericytes and identified key candidate targets. By correlating pericyte behavior with molecular alterations, this study demonstrates that pericytes play a critical role in GBM angiogenesis, providing a foundation for pericyte‐targeted therapies.

## 2 MATERIALS AND METHODS

### 2.1 Mice

We established two genetically engineered mouse models for this study^1^: Pdgfrb‐tdTomato reporter mice (PT mice) by crossing Ai14 reporter mice (Jackson Laboratories, stock #007914) with *Pdgfrb‐P2A‐CreER* ^*T2*^ mice (Jackson Laboratories, stock #030201), and^2^ Pdgfrb^+^ cell ablation mice through subsequent breeding of PT mice with *ROSA‐DTA* mice (Jackson Laboratories, stock #009669). All mice were congenic on the C57BL/6J genetic background. Both male and female mice were used for all experiments without preference. Genotypes of all mice were determined using PCR analyses of tail or toe genomic DNA with appropriate primers. All primers were ordered from Sangon Biotech based on the sequences provided by the Jackson Laboratory's official website.

All mice used in this study were Specific Pathogen Free (SPF)‐grade and obtained from The Jackson Laboratory. The animals were housed in barrier facilities under strictly controlled environmental conditions, including maintained temperature (22–25°C) and humidity (40%–60%), and a 12‐h light/dark cycle. Animals were housed with a maximum of 5 mice per cage and provided with ad libitum access to food and water. All experimental procedures were performed in accordance with institutional guidelines and approved by the Institutional Animal Care and Use Committee (IACUC) of the Chinese Academy of Medical Sciences (Approval No.: GR24001).

### 2.2 Tumor cell culture and lentivirus infection

The GL261 murine glioblastoma cell line was obtained from ATCC and cultured in RPMI‐1640 medium supplemented with 10% FBS. To establish stable reporter cell lines, GL261 cells were transduced with lentiviral vectors at a MOI of 100. For GL261‐CFP cells, the culture medium was replaced after 16 h of infection with CFP‐encoding lentivirus, followed by selection with 500 μg/mL G418 for 72 h. Similarly, GL261‐Luc cells were generated by lentiviral transduction of the luciferase gene under identical infection conditions (MOI = 100), with subsequent selection using 0.7 μg/mL puromycin.

### 2.3 Orthotopic GBM model

Two orthotopic glioblastoma (GBM) mouse models were established for distinct imaging applications. For bioluminescence imaging (IVIS), GL261‐Luc cells were stereotactically implanted into the left caudate putamen (CPu) at coordinates 1.5 mm lateral (left), 1.0 mm anterior to bregma, and 3.5 mm below the skull surface (*n* = 4 mice per group). For two‐photon intravital microscopy imaging, cranial window preparation was performed in PT mice (*n* = 6) under anesthesia induced by intraperitoneal injection of ketamine (80 mg/kg) and xylazine (10 mg/kg). Following skull and dura mater removal, a 6‐mm circular cover glass was implanted. Four weeks post‐window preparation, 2 μL of GL261‐CFP cell suspension (5 × 10^3^ cells) was injected into the right parietal cortex using a 10 μL Hamilton microsyringe with a 2‐pt style needle. The stereotactic injection coordinates were carefully determined as 1.0 mm anterior to bregma (aligned with the midpoint of the exposed superior sagittal sinus) and 1.5 mm lateral to the midline, with special attention paid to avoiding major blood vessels to minimize hemorrhage risk. Mice used in this in vivo two‐photon imaging study were 6 weeks old at cranial window implantation and 8 weeks old during GL261 tumor cell inoculation.

### 2.4 Two‐photon intravital imaging

A Leica TCS SP8 DIVE confocal microscope equipped with a Chameleon Ultra laser (680–1300 nm) and a 25× water‐immersion objective (NA 0.95) was used to acquire images. The acquisition depth of a single image ranged from 0 to 300 μm with a z‐interval of 3 μm. The excitation wavelength for FITC‐dextran and TdTomato was 975 nm, and that for GL261‐CFP was 860 nm, with a resolution of 1024 × 1024 pixels. To visualize the cerebral blood vessels, 0.2 mL of 10 mg/mL FITC‐dextran (molecular weight of 2 × 10^6^, green) was injected into the tail vein of the mouse. The emission wavelengths were 570–640 nm for TdTomato, 500–550 nm for FITC‐dextran, and 450–490 nm for GL261‐CFP tumor cells. Isoflurane concentration was titrated (0.8%–2.0%) based on respiratory rate, with laser power minimized to reduce phototoxicity. The laser power was also limited to the lowest percentage to minimize phototoxicity.

### 2.5 Statistical analysis

Image processing and quantitative analysis were performed using Imaris software. The Slice module measured vessel diameter and tumor major axis, while the Filament Tracer module analyzed vascular branching point distances, pericyte distribution along vessels, and pericyte protrusion lengths. Tumor volume (*V*) was calculated using the formula:

$$V=4/\text{3π}{\left(d/2\;\right)}^{3},$$

where *d* represents the tumor diameter.

For statistical analysis, GraphPad Prism 8 (version 10.4.1) was employed. No predefined statistical methods were used to determine sample size; instead, randomly selected regions of interest were analyzed. Each statistical value was derived from more than 10 independent samples. The Mann–Whitney test (two‐tailed, non‐parametric) was applied to all image‐derived datasets, with significance thresholds defined as: *p < 0.05, **p < 0.01, and ***p < 0.001.

### 2.6 Pericyte proteomic profiling

GBM or brain was dissected in PBS. The tissue was cut into small pieces using scissors. Subsequently, the homogenate and digestion buffer were transferred into a 15‐ml centrifuge tube. The mixture was incubated at 37°C with gentle stirring for 10 minutes, after which 5 mL of wash buffer (FACS buffer) was added. Homogenates were filtered through a 70‐μm strainer, centrifuged (400 *g*, 5 min), resuspended in FACS buffer, and the supernatant was carefully discarded. The pellet was resuspended in 500 μL of FACS buffer. The sample was kept on ice throughout the process to maintain its stability prior to FACS sorting.

tdTomato^+^ Pdgfrb^+^ cells were isolated using a BD FACSAria III, flash‐frozen in liquid nitrogen, and pulverized. Protein concentration was quantified via the BCA assay. Peptides were fractionated by high‐pH reverse‐phase chromatography and analyzed on a Q‐Exactive HF LC–MS/MS system.

### 2.7 Data quality control and analysis

The DIA data were processed using the DIA‐NN search engine (v.1.8). Tandem mass spectra were searched against Mus_musculus_10090_SP_20230103.fasta (17 132 entries) concatenated with reverse decoy database. Trypsin/P was specified as cleavage enzyme allowing up to 1 missing cleavage. Excision on N‐term Met and carbamidomethyl on Cys were specified as fixed modifications. FDR was adjusted to <0.01.

### 2.8 Bioinformatics methods

#### 2.8.1 Normalization

The expression levels of genes were calculated using the CPM (counts per million) metric from the edgeR (v3.16, https://bioconductor.org/packages/release/bioc/html/edgeR.html) R package.

#### 2.8.2 Differential expression and cluster analysis

Differentially Expressed Genes (DEGs) between different sample groups were identified using the edgeR (v3.16) R package. The trimmed mean of M values (TMM) method was applied for normalization. Genes with |log2FC| ≥ 1.5 and *p* value ≤0.01 were considered significantly differentially expressed. Hierarchical clustering was performed using the pheatmap package (https://cran.r‐project.org/web/packages/pheatmap/index.html) with default settings, employing Spearman's correlation distance. Optimal clustering of all DEGs identified through pairwise comparisons was achieved using the k‐means method.

Protein expression data were analyzed and visualized using the OmicShare tools platform (https://www.omicshare.com/tools/).

#### 2.8.3 Functional annotation and enrichment analysis

Pathway enrichment analysis was conducted using the Blast‐KOALA tool from the Kyoto Encyclopedia of Genes and Genomes (KEGG) database, and pathway modules were further explored using the KEGG mapper tool. Gene Ontology (GO) annotations and sequence information for all DEGs were retrieved from the UniProt database using the UniProt Retrieve/ID mapping tool. GO enrichment analysis was performed using WEGO, and the results were visualized using the ggplot2 package in R.

## 3 RESULTS

### 3.1 Pericyte ablation exerts biphasic effects on GBM growth: Early suppression followed by late‐stage acceleration

**FIGURE 1:**
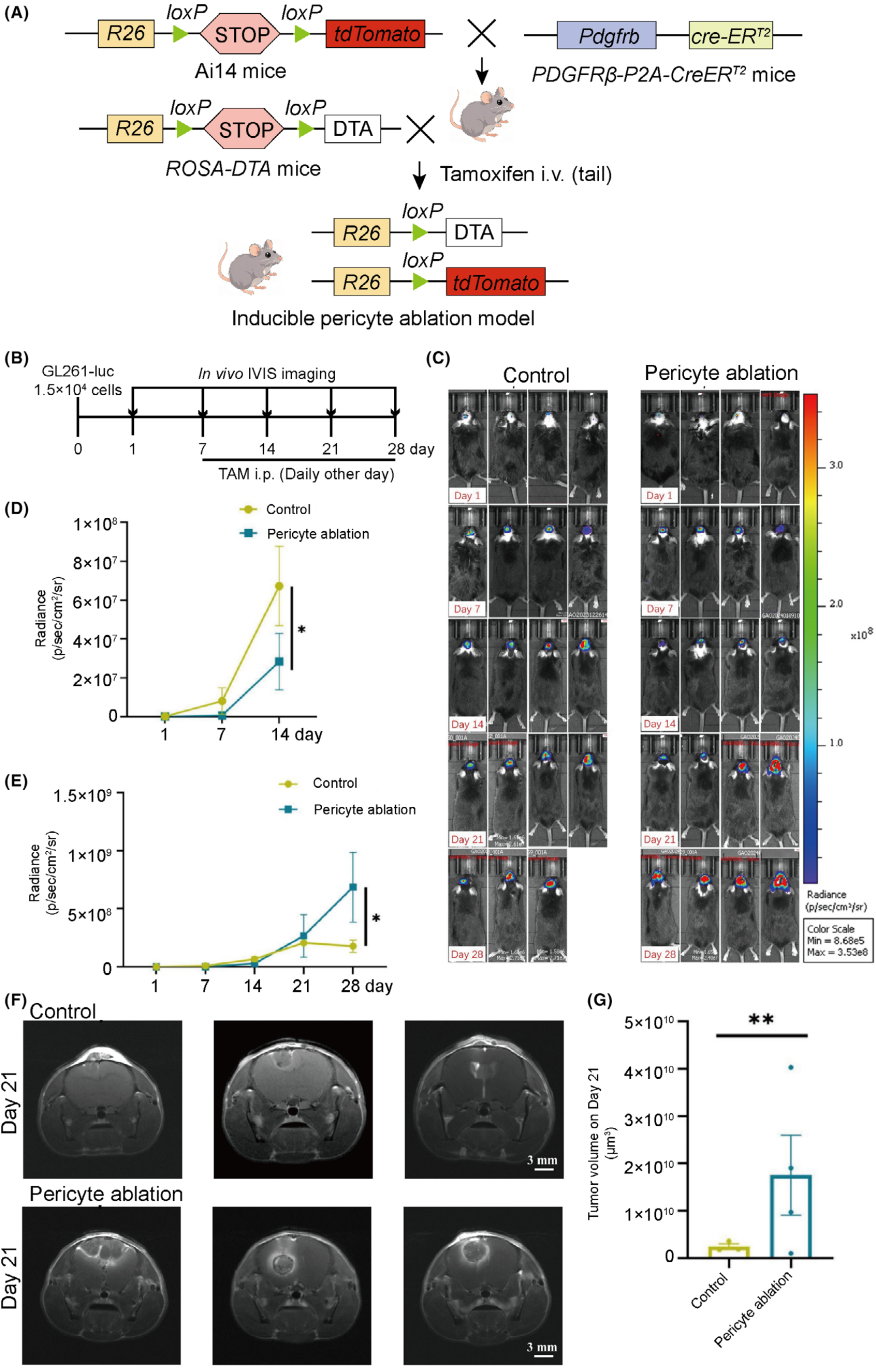
Pdgfrb^+^ cell Ablation Exerts Biphasic Effects on GBM Growth: Early Suppression Followed by Late‐Stage Acceleration. (A) Strategy for conditional labeling and ablation model construction of Pdgfrb^+^ cells. (B) Timeline for orthotopic brain tumor inoculation and in vivo small animal imaging observation. (C–E) In vivo small animal fluorescence imaging (C, scale bar: 3 mm) and its quantification plots (D, E). (F–G) MRI of mouse brains (F) and tumor volume plots (G; **p < 0.01, error bars = SEM).

To investigate the functional role of pericytes in glioblastoma (GBM), We first established and validated a tamoxifen‐inducible, Pdgfrb^+^ cell‐specific ablation mouse model (PT mice × ROSA‐DTA mice) through genotyping prior to experimental use (Figure S1A–C). In this model, Pdgfrb^+^ cells could be selectively ablated through tamoxifen‐inducible, Cre‐mediated diphtheria toxin expression while simultaneously being labeled with tdTomato (Figure 1A). In a double‐blind experimental design, both control and ablation groups received intracranial implantation of 1.5 × 10^4^ GL261‐luc cells (Day 0). Bioluminescence imaging on Day 1 confirmed comparable tumor cell engraftment between groups, with no significant differences in tumor proliferation observed by Day 7 under baseline conditions (Figure 1B). Given the stem‐like properties of pericytes, tamoxifen (TAM) was administered every other day starting from Day 7 to systematically ablate newly generated pericytes (Figure 1B).

Notably, in vivo imaging system (IVIS) analysis revealed a biphasic effect of pericyte ablation: transient suppression of early‐stage GBM proliferation followed by accelerated tumor growth in intermediate/late stages (Figure 1C–E). Quantitative analysis demonstrated that Pdgfrb^+^ pericyte ablation ultimately promoted GBM progression, with magnetic resonance imaging (MRI) confirming significantly larger tumor volumes in the ablation group compared to controls by Day 21 (Figure 1F,G). Hematoxylin and Eosin (H&E) staining of tumor‐bearing brain sections demonstrated characteristic glioblastoma histopathological features, including hypercellularity, nuclear atypia, and pseudopalisading necrosis, as shown in Figure S1D. These results demonstrate that during the early stage of GBM, the presence of pericytes facilitates tumor growth.

### 3.2 Establishment of a mouse model for in vivo tracking of Pdgfrb^+^ cell dynamics

**FIGURE 2:**
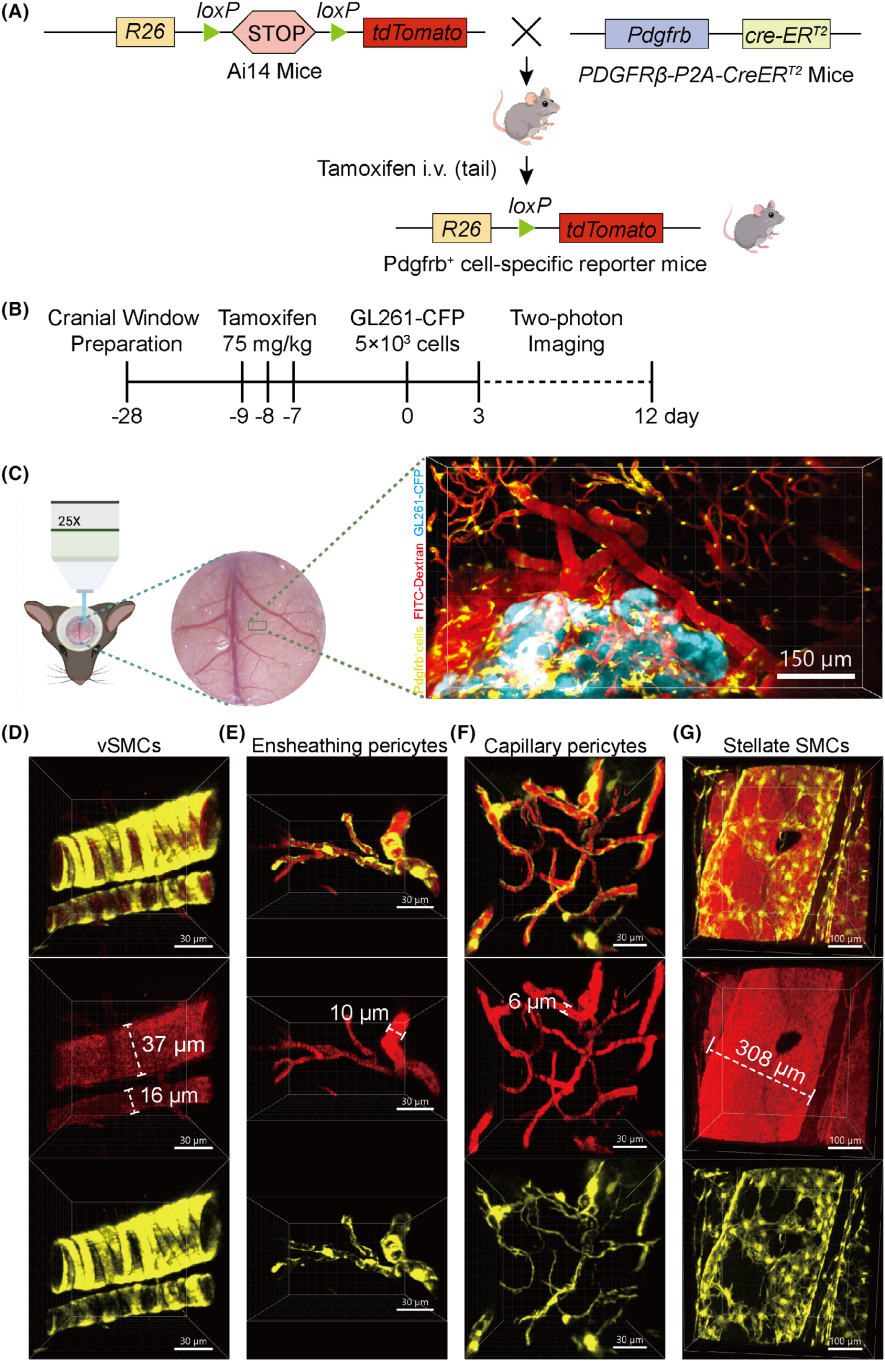
Establishment of a Mouse Model for In Vivo Tracking of Pdgfrb^+^ Cell Dynamics. (A) Strategy for conditional labeling of Pdgfrb^+^ cells. (B) Timeline for orthotopic brain tumor inoculation and two‐photon fluorescence microscopy observation. (C) longitudinal two‐photon imaging platform for spatiotemporal tracking of vascular Pdgfrb^+^ cells dynamics in GBM models. (D–G) Representative images of different Pdgfrb^+^ cell subtypes: VSMCs on arteries (D), ensheathing pericytes on pre‐capillary arterioles (E), capillary pericytes on capillaries (F), and stellate SMCs on veins (G).

To observe the dynamic behavior of Pdgfrb^+^ cells during the early stages of GBM, we constructed a Pdgfrb^+^ cell‐specific reporter mouse model (Figure 2A) and designed a rigorous experimental workflow (Figure 2B). Using the time of tumor cell injection as day 0, cranial window surgery was performed on the model mice 28 days in advance. Tamoxifen was administered via intraperitoneal injection for three consecutive days from day −9 to day −7 to induce tdTomato reporter gene expression and label Pdgfrb^+^ cells. On the day of tumor inoculation, the old cranial window was carefully removed to minimize brain tissue damage and inflammatory responses. GL261‐CFP cells were then precisely injected into the parietal cortex of each mouse at a cell dose of 5 × 10^3^ using a stereotactic apparatus, and a new cranial window was installed. Starting from day 3, daily intravital two‐photon microscopy imaging was performed with the same tumor as the central field of view. Images were optimized through pseudocolor processing: blood vessels were labeled red, Pdgfrb^+^ cells appeared yellow, and tumors were shown in blue. As shown in Figure 2C, by using a two‐photon microscope, we obtained three‐dimensional (3D) images of tumors, blood vessels, and Pdgfrb^+^ cells at multiple time points. This approach enabled direct in vivo tracking and visualization of the angiogenesis process within GBMs.

By using cell morphology and vascular branch order criteria, we identified four subtypes of Pdgfrb^+^ cells in vivo in normal brain regions^25^, ^26^: circular vascular‐associated smooth muscle cells (vSMCs) on arteries, ensheathing pericytes on pre‐capillary arterioles, capillary pericytes (with elongated processes extending longitudinally along capillaries) on capillaries, and stellate SMCs on veins (Figure 2D–G). These Pdgfrb^+^ cells all belong to mural cells.

In summary, the development of this mouse model successfully captured the progressive structural reorganization of GBM‐associated vascular networks, thereby enabling real‐time, longitudinal tracking of Pdgfrb^+^ cells dynamics within the early GBM microenvironment. Concurrently, the identification of Pdgfrb^+^ cell subtypes in normal brain tissue establishes a critical baseline for comparative analysis of pathological vascular remodeling in glioblastoma.

### 3.3 Pericyte‐dominated Pdgfrb^+^ cell dynamics and vascular remodeling from vessel co‐option to angiogenesis in GBM

**FIGURE 3:**
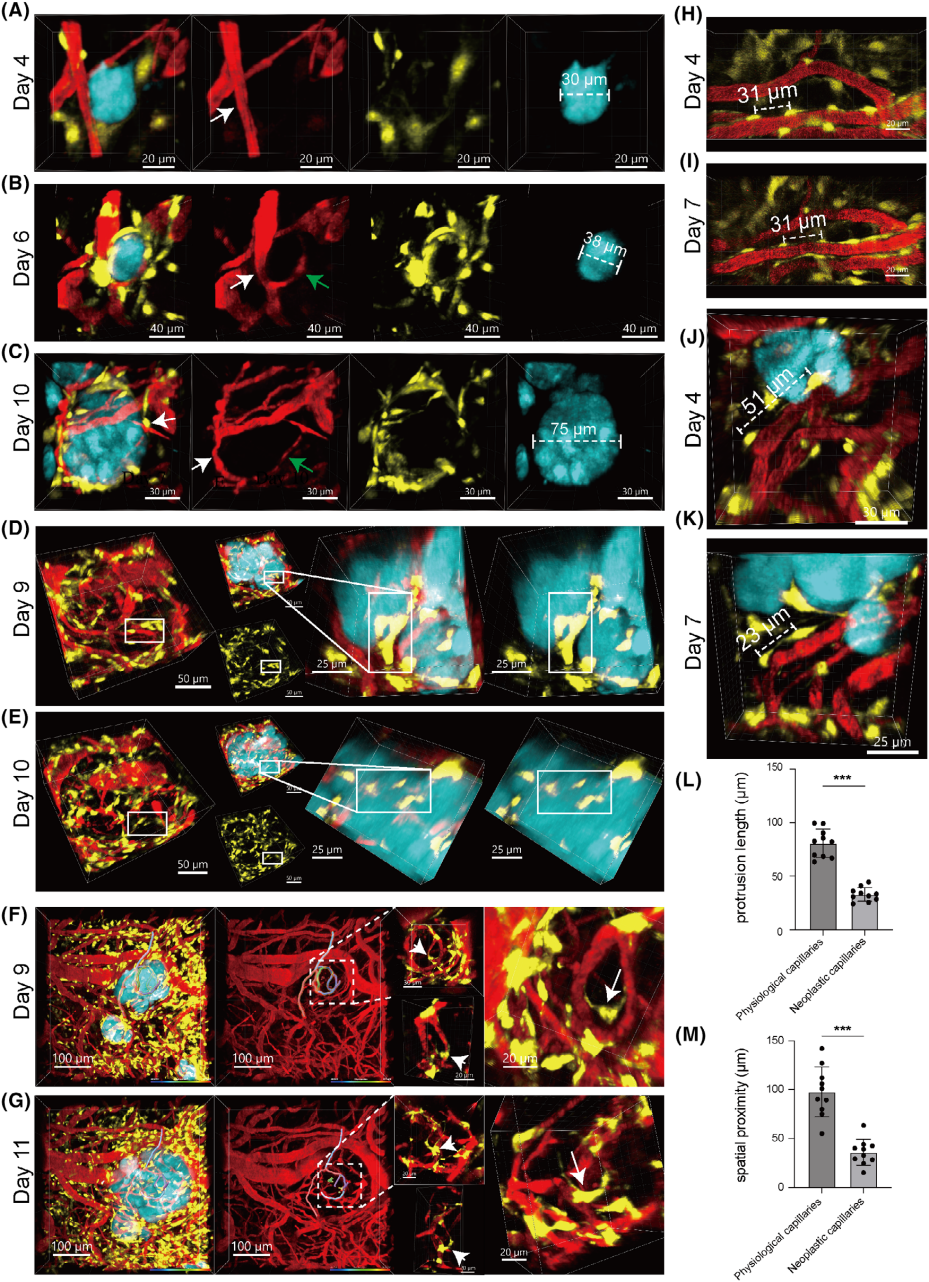
Pericyte‐Dominated Pdgfrb^+^ Cell Dynamics and Vascular Remodeling from Vessel Co‐option to Angiogenesis in GBM. (A) Vascular co‐option without neovascularization phase, scale bar: 30 μm. (B, C) Angiogenic sprouts from native vessels (B, scale bar: 40 μm) and vascular bridging (C, scale bar: 30 μm). White arrows: Pre‐existing blood vessels; Green arrows: Newly formed blood vessels. (D, E) Images within the white box show the spatial distribution changes of Pdgfrb^+^ cells in the brain‐GBM border region over 24 h from dual perspectives (Scale bars: Left three panels, 50 μm; right two panels, 25 μm). (F, G) Images show the dynamic distribution changes of Pdgfrb^+^ cells in the GBM core region over 48 h from three perspectives (Scale bars: Left two panels, 100 μm; right three panels, 20 μm). (H–K) Representative images of pericytes on the same blood vessels in regions distant from (H, I) and adjacent to (J, K) the tumor, captured on days 4 and 7 post‐tumor inoculation. (L–M) Quantitative analysis of pericyte process length and spatial proximity between pericytes along blood vessels (***p < 0.001).

During our observation period (Days 3–12), Pdgfrb^+^ cells at the brain‐GBM interface and within tumors predominantly exhibited pericyte morphology (Figures 3A–G and S2A–E,H,I,M), indicating that pericytes are the dominant Pdgfrb^+^ cell population in the GBM microenvironment. Early tumor progression was characterized by vessel co‐option, where tumor cells hijacked pre‐existing host vasculature, resulting in irregular lumens and fluctuating diameters (Figures 3A and S1E). At this stage, sparse pericytes accumulated at the tumor periphery (Figure 3A), with no detectable vascular sprouts, supporting vessel co‐option as the initial nutrient acquisition mechanism.

As tumors grew, the mass effect displaced host vasculature at the invasive front (Figure S1F–H), while angiogenesis occurred in the tumor core (Figure S1H), establishing neovascularization as the primary vascularization mode in advanced GBMs. We captured the dynamic interplay between pericytes and angiogenesis: at a tumor diameter of 38 μm, vascular sprouts emerged precisely in pericyte‐enriched zones, accompanied by further pericyte recruitment (Figure 3B,C). At 75 μm, host vessels were displaced by mass effect, while neovessels formed branches and were integrated into the tumor, creating interconnected networks between new and displaced vasculature. Notably, pericytes consistently localized to neovessel branch points (white arrow, Figure 3C), establishing a spatial correlation between pericyte positioning and angiogenic sites and demonstrating pericyte‐mediated vascular remodeling.

As shown in Figure S2A,B, a single host vessel was remodeled into a capillary network over three days. Notably, pericytes recruited at the tumor edge formed aggregated clusters, while intratumoral pericytes were diffusely distributed across the neovascular network. Do pericytes on intratumoral vascular networks originate from those recruited at the tumor edge?

We performed detailed analyses of local pericyte dynamics. First, using Imaris software, we overlapped two 3D images captured 1 day apart, using vascular branch points and the tumor boundary as reference systems, and performed spatial registration by integrating the morphological features of target pericyte clusters. 3D overlap analysis of cell clusters within the white boxes in Figure 3D,E revealed that their geometric configurations and movement trajectories exhibited spatiotemporal continuity consistent with the pathological process, confirming that they represented the same cell cluster. This indicates that the diffusely distributed pericytes on neovessels in Figure 3E migrated from the clustered pericytes at the tumor edge shown in Figure 3D one day prior. Collectively, our results demonstrate that tumor‐edge‐recruited pericytes can migrate and integrate into neovessels.

Next, using the Filament Tracer module in Imaris software, we performed 3D reconstruction of blood vessels to track the dynamic behavior of the same vessel and its adjacent pericytes. In the GBM core, we captured the dynamic process of individual pericytes transitioning from a free‐floating state outside blood vessels to attachment onto the vessel wall (Figure 3F,G). This process aligns with the theoretical model where endothelial cells first form luminal structures, followed by pericyte recruitment.

What is the significance of pericyte coverage for GBM neovessels? We observed that pericytes predominantly localized at branch points of neovessels (Figure S2C–E), a distribution pattern analogous to normal vascular development. We therefore hypothesize that their functions are consistent with those in normal vascular development, i.e., pericyte coverage is associated with the maturation and stabilization of GBM neovessel lumens, thereby supporting tumor growth.

Finally, we characterized pathological changes in GBM‐associated pericytes compared to those in normal brain tissue. As shown in Figure 3H,I, pericytes in physiological states stably localize on vessel walls, whereas GBM‐associated pericytes (Figure 3J,K) exhibit active migration. Morphological and density analysis of pericytes using Imaris software revealed that GBM‐associated pericytes had significantly shorter process lengths (Figure S2F vs. S2G, quantified in Figure 3L) but exhibited significantly decreased inter‐pericyte distance compared to normal brain pericytes (quantified in Figure 3M), suggesting active proliferation of GBM‐associated pericytes and potentially distinct expression of proteins influencing cell morphology.

Additionally, the native brain capillary plexus exhibited substantial heterogeneity in branch‐point spacing, ranging from 21.5 to 97.3 μm (Figure S2H, quantified in Figure S2J). In contrast, tumor neovessels displayed significantly narrowed variability in branch‐point spacing (18.1–60.4 μm), indicative of structural simplification in the tumor microenvironment (Figure S2I, quantified in Figure S2J). Furthermore, neovessels demonstrated significantly luminal distension (median: 10.55 μm) compared to normotypic capillaries (median: 6.365 μm; Figure S2K).

### 3.4 Proteomic molecular characterization of mouse brain and GBM Pdgfrb^+^ cells

To systematically investigate the molecular perturbations of pericytes in the brain tumor microenvironment, we employed LC–MS/MS‐based high‐throughput proteomic analysis. First, Pdgfrb^+^ cell populations were specifically enriched from mouse normal brain tissue and tumor tissue via fluorescence‐activated cell sorting (FACS), designated as GBM‐associated Pdgfrb^+^ cells (Mural_T) and normal brain‐derived Pdgfrb^+^ cells (Mural_N), respectively. These results partially reflect GBM‐induced reprogramming of pericytes.

**FIGURE 4:**
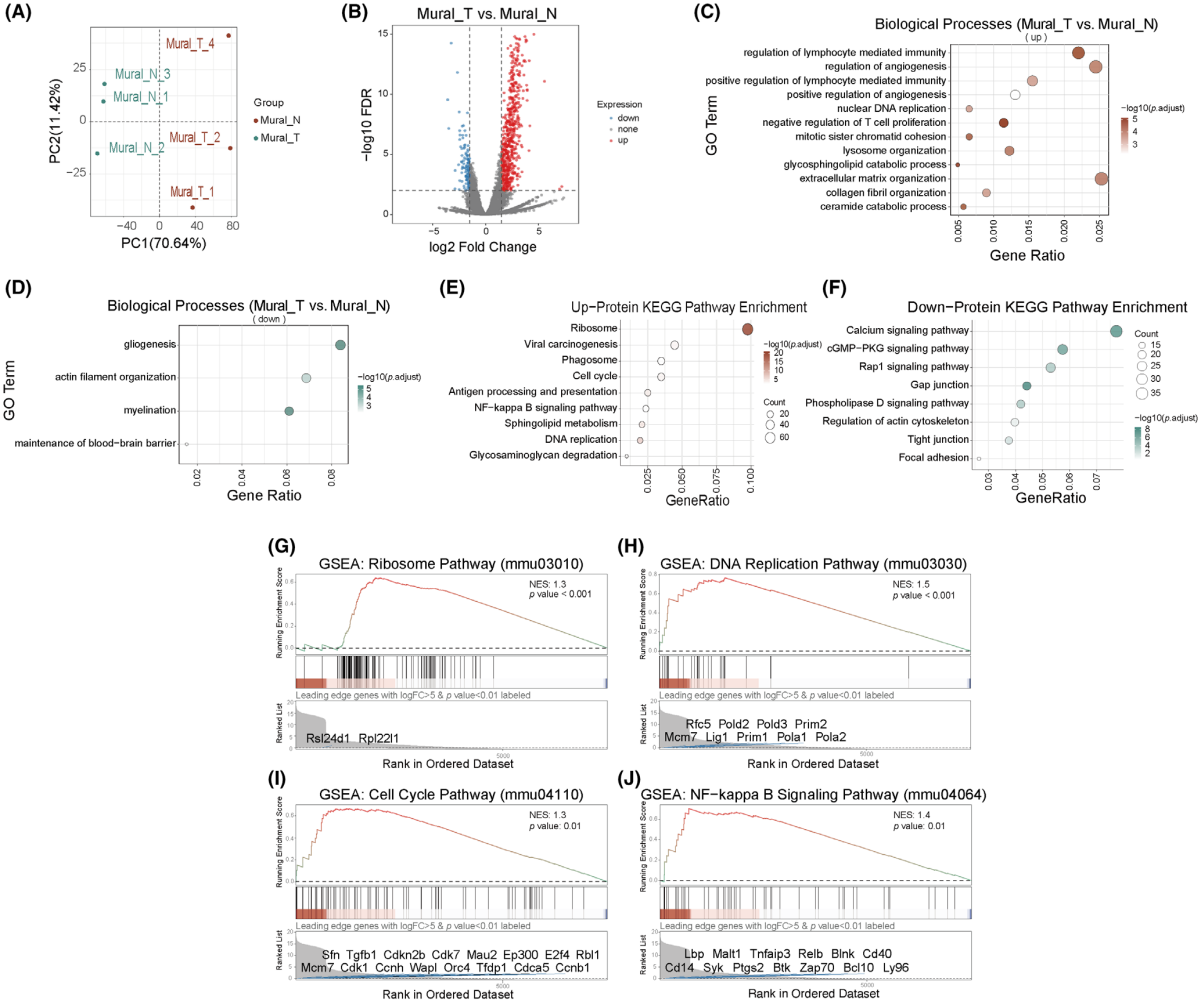
Proteomic molecular characterization of mouse brain and GBM Pdgfrb^+^ cells. (A) Scatter plot based on principal component analysis (PCA). (B) Volcano plot of differentially expressed proteins. (C, D) GO enrichment analysis plots for upregulated (C) and downregulated (D) proteins. (E, F) KEGG pathway enrichment analysis plots for upregulated (E) and downregulated (F) proteins. (G–J) Gene set enrichment analysis (GSEA) plots for the ribosome pathway (G), DNA replication pathway (H), cell cycle pathway (I), and NF‐κB signaling pathway (J).

Proteomic sequencing identified 6783 high‐confidence proteins (Figure S3A). Raw data preprocessing strictly followed quality control standards, including peptide length distribution (Figure S3B) and peptide count distribution (Figure S3C), both meeting quality requirements. To ensure data comparability, we normalized the expression matrix using counts per million (CPM) normalization (Figures S3D,E). Principal component analysis revealed significant separation between tumor and normal groups along the PC1 dimension (explaining 70.64% of variance), indicating fundamental proteomic differences (Figure 4A). This finding was further validated by a correlation heatmap (Figure S3F), where intra‐group Pearson correlation coefficients (*r* > 0.89) were significantly higher than inter‐group coefficients (*r* < 0.8), confirming tumor microenvironment‐induced molecular signatures.

Hierarchical clustering analysis (Figure S3G) combined with differential expression analysis (FDR <0.01, |log2FC| >1.5) showed 1286 significantly upregulated and 140 downregulated proteins in Mural_T compared to normal pericytes. Global differential protein expression was visualized in a volcano plot (Figure 4B), highlighting molecular reprogramming in Mural_T versus Mural_N. This highly asymmetric regulation (upregulated proteins outnumbering downregulated by ~9.2‐fold) strongly suggests profound GBM‐induced molecular reprogramming in pericytes, potentially involving activation of pro‐tumorigenic pathways and suppression of homeostatic functions.

GO enrichment analysis revealed significant enrichment of pro‐proliferative and pro‐angiogenic processes in Mural_T, including “mitotic sister chromatid cohesion,” “nuclear DNA replication,” “positive regulation of angiogenesis,” and “collagen fibril organization” (Figure 4C). In stark contrast, biological processes essential for vascular homeostasis—such as “actin filament assembly” and “blood‐brain barrier maintenance”—were systematically downregulated (Figure 4D).

KEGG enrichment analysis further elucidated systematic functional remodeling in pericytes (Figure 4E,F). Enrichment of ribosome biogenesis, cell cycle, and DNA replication pathways validated acquired proliferative capacity in Mural_T. Additionally, coordinated activation of glycosaminoglycan degradation, sphingolipid metabolism, and NF‐κB signaling pathways sustained a pro‐inflammatory and pro‐proliferative microenvironment. Conversely, suppression of calcium signaling and cGMP‐PKG signaling pathways may correlate with pericyte‐mediated vascular dysregulation. Widespread downregulation of cell‐junction pathways (e.g., gap junctions, tight junctions, focal adhesions) explained aberrantly enhanced pericyte migration.

Gene set enrichment analysis (GSEA) demonstrated a cascade regulatory network involving ribosome (Figure 4G), cell cycle (Figure 4H), and DNA replication pathways (Figure 4I). NF‐κB signaling was also significantly activated (Figure 4J). Leading genes in these pathways (logFC >5, *p* value <0.01) represent potential therapeutic targets for GBM angiogenesis intervention (Figure 4G–J).

In summary, GBM induces functional transformation of pericytes through molecular hijacking—activating pro‐tumor proliferative/inflammatory networks while dismantling vascular homeostatic programs. The markedly imbalanced proteomic landscape (9.2:1 ratio) underscores the potential of pericytes as key therapeutic targets.

### 3.5 Conserved molecular reprogramming of pericytes in human GBM: ECM and pro‐migratory targets

To validate whether the above findings are conserved in human GBM‐associated pericytes, we performed GO and KEGG enrichment analyses on RNA sequencing data derived from human brain and GBM pericytes. Data were sourced from the GSE242044 scRNA‐seq dataset, which includes 3 surgically resected GBM samples and 4 matched adjacent normal brain tissue samples. Pericytes were identified as cells positive for pan‐mural cell markers, including PDGFRB, notch receptor 3 (NOTCH3), hypoxia‐inducible gene domain 1β (HIGD1B), and regulator of G‐protein signaling 5 (RGS5), and negative for smooth muscle cell (SMC) markers, such as transgelin (TAGLN), actin alpha 2, smooth muscle (ACTA2), and myosin heavy chain 11 (MYH11).^27^

**FIGURE 5:**
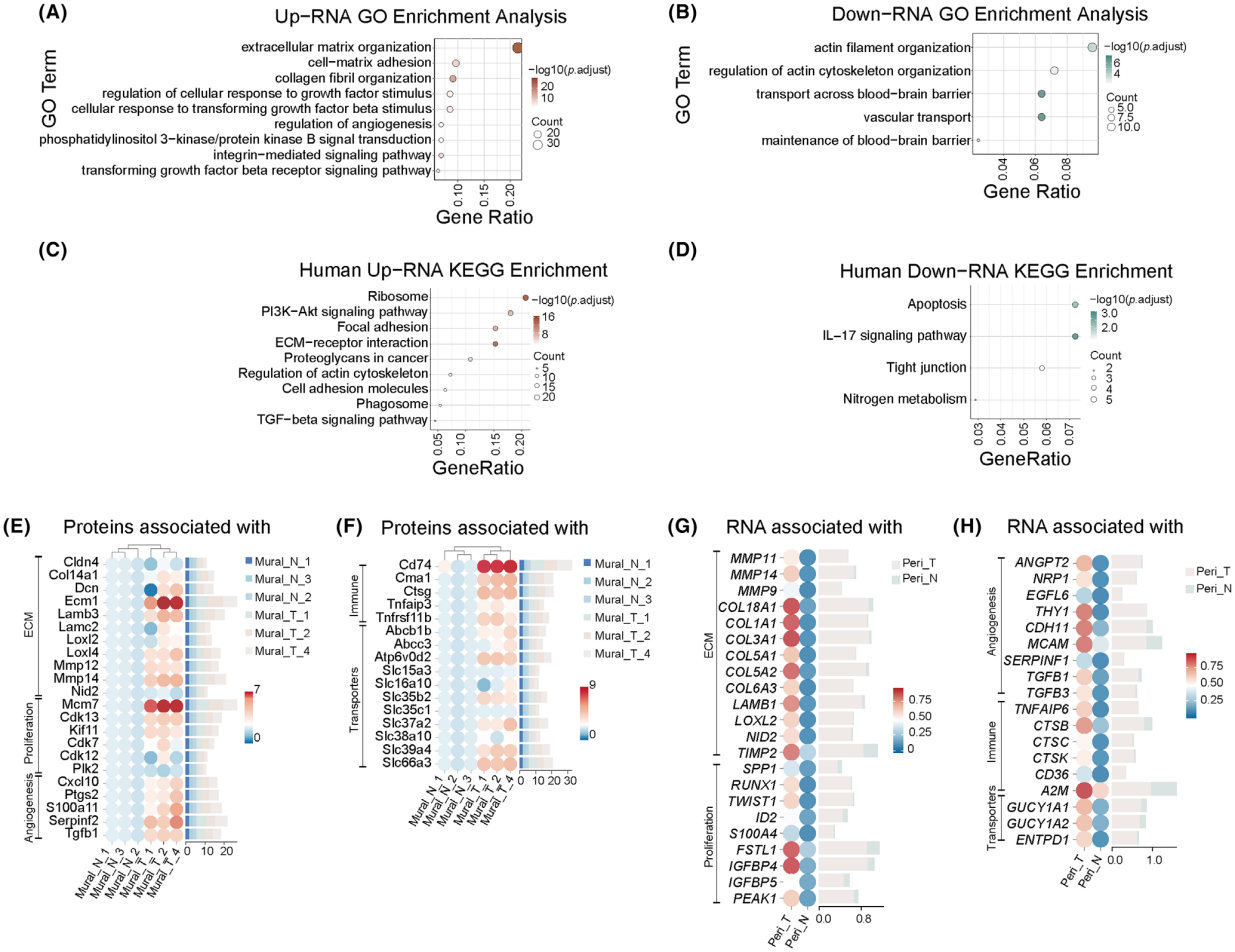
Conserved Molecular Reprogramming of Pericytes in Human GBM: ECM and Pro‐migratory Targets. (A, B) GO enrichment analysis plots for upregulated (A) and downregulated (B) RNAs. (C, D) KEGG pathway enrichment analysis plots for upregulated (C) and downregulated (D) RNAs. (E–H) Expression of relevant proteins in mouse samples (E, F) and expression of relevant RNA in human samples (G, H).

Analysis revealed conserved alterations in pathways related to cell proliferation, cell motility, vascular homeostasis, and inflammatory response—originally identified at the protein level in mouse GBM Pdgfrb^+^ cells—at the RNA level in human GBM pericytes (Figure 5A–D). Notably, results from both human and mouse models demonstrated broad enrichment of extracellular matrix (ECM)‐, proliferation‐, angiogenesis‐, immune‐, and transport‐related proteins (Figure 5E–H). ECM‐related proteins likely play key roles in the enhanced migratory activity of Mural_T cells. Critically, dysregulation of core molecules including MMP14, LOXL2, NID2, and transforming growth factor beta 1 (TGFB1) was highly conserved across species, suggesting their potential as promising therapeutic candidate targets. (Figure 5E,G).

## 4 DISCUSSION

Our study uncovers a spatiotemporally orchestrated hijacking of pericytes in glioblastoma, wherein these vascular guardians are coercively transformed into tumor accomplices. The biphasic effects of pericyte ablation reveal their dualistic roles as structural stabilizers during initial vascular co‐option versus pro‐tumorigenic effectors in advanced angiogenesis. This temporal duality underscores the peril of untargeted interventions, as early ablation may disrupt homeostatic pericytes that transiently restrain tumor expansion.

Longitudinal intravital imaging directly captures the dynamic migration and integration of tumor‐edge‐recruited pericytes into nascent vascular networks. These cells exhibit morphological plasticity and strategically localize at neovessel branch points to scaffold angiogenic switching—a process fueled by molecular reprogramming rather than mere activation. The discovery of free‐floating pericytes incorporating into vessel walls provides unprecedented evidence of their role in neovessel maturation.

At the molecular level, proteomics reveals activation of proliferative‐angiogenic machineries and systematic erosion of homeostatic functions. The marked imbalance toward pro‐tumor pathways reflects GBM's exploitation of pericytes as metabolic and structural co‐conspirators.

Strikingly, human GBM pericytes recapitulate this reprogramming, with conserved dysregulation of key extracellular matrix remodeling components. Their cross‐species conservation positions them as high‐priority therapeutic targets.

Despite these advances, limitations persist regarding tumor model heterogeneity and mechanistic validation of specific pathways. The origin of pericytes on GBM neovessels remains controversial, with proposed sources including glioma stem cell differentiation,^28^ endogenous vascular precursors,^29^ or abnormal reprogramming of host‐derived normal cells by the microenvironment.^27^ In this study, pericytes in GBM neovessels were exclusively traced to host‐derived Pdgfrb^+^ cells. Therapeutically, our data advocate for timing‐stratified strategies: inhibiting pericyte proliferation or migration in the early stage of disease to suppress angiogenesis and vascular maturation, while selectively targeting inflammation‐related drivers or promoting vascular homeostasis in the late stage. Additionally, developing drug delivery systems targeting Pdgfrb^+^ cells at the brain‐GBM interface can leverage the region's active lipid metabolism to optimize local drug retention and efficacy.^30^, ^31^, ^32^, ^33^ Such approaches enable precise inhibition of angiogenesis while circumventing drug penetration barriers caused by hypoxia and fibrosis in the tumor core, eliminating the reliance on deep intratumoral drug administration.^30^, ^34^, ^35^, ^36^, ^37^ Validating these approaches in patient‐derived models will be essential to translate the biology of pericyte hijacking into clinical breakthroughs.

## AUTHOR CONTRIBUTIONS

**Qinghong Wang:** Conceptualization; formal analysis; investigation; methodology; software; validation; visualization; writing – original draft. **Chengyan Ma:** Formal analysis; investigation; methodology; visualization; writing – original draft. **Xinpei Wang:** Investigation; methodology; validation; writing – review and editing. **Mengyuan Li:** Investigation; methodology; writing – review and editing. **Xingjiu Yang:** Investigation; methodology; writing – review and editing. **Ran Gao:** Conceptualization; funding acquisition; investigation; methodology; project administration; resources; supervision; validation; writing – review and editing.

## FUNDING INFORMATION

The authors gratefully acknowledge support for this work from the National Key Research and Development Program of China (grant numbers 2022YFF0710700), the Non‐profit Central Research Institute Fund of Chinese Academy of Medical Sciences (2023‐PT180‐01).

## CONFLICT OF INTEREST STATEMENT

The authors declare no competing interests. Ran Gao is an editorial board member of AMEM and a coauthor of this article. To minimize bias, she was excluded from all editorial decision making related to the acceptance of this article for publication.

## ETHICS STATEMENT

All experimental procedures involving animals were approved by the Institutional Animal Care and Use Committee (IACUC) of the Chinese Academy of Medical Sciences (Approval No. GR24001).

## Supporting information


Figure S1.
