# Supplementary material for: Insight into pericytes in glioblastoma angiogenesis: In vivo tracking by two‐photon microscopy and proteomic profiling
Source: Animal Model Exp Med. 2025 Aug 7;8(9):1688–99. doi: 10.1002/ame2.70073 (PMC12531104; doi:10.1002/ame2.70073)
Supplement: Supplementary file 1 — Figure S1. [file AME2-8-1688-s001.docx]

**Supplementary Figure 1**

**
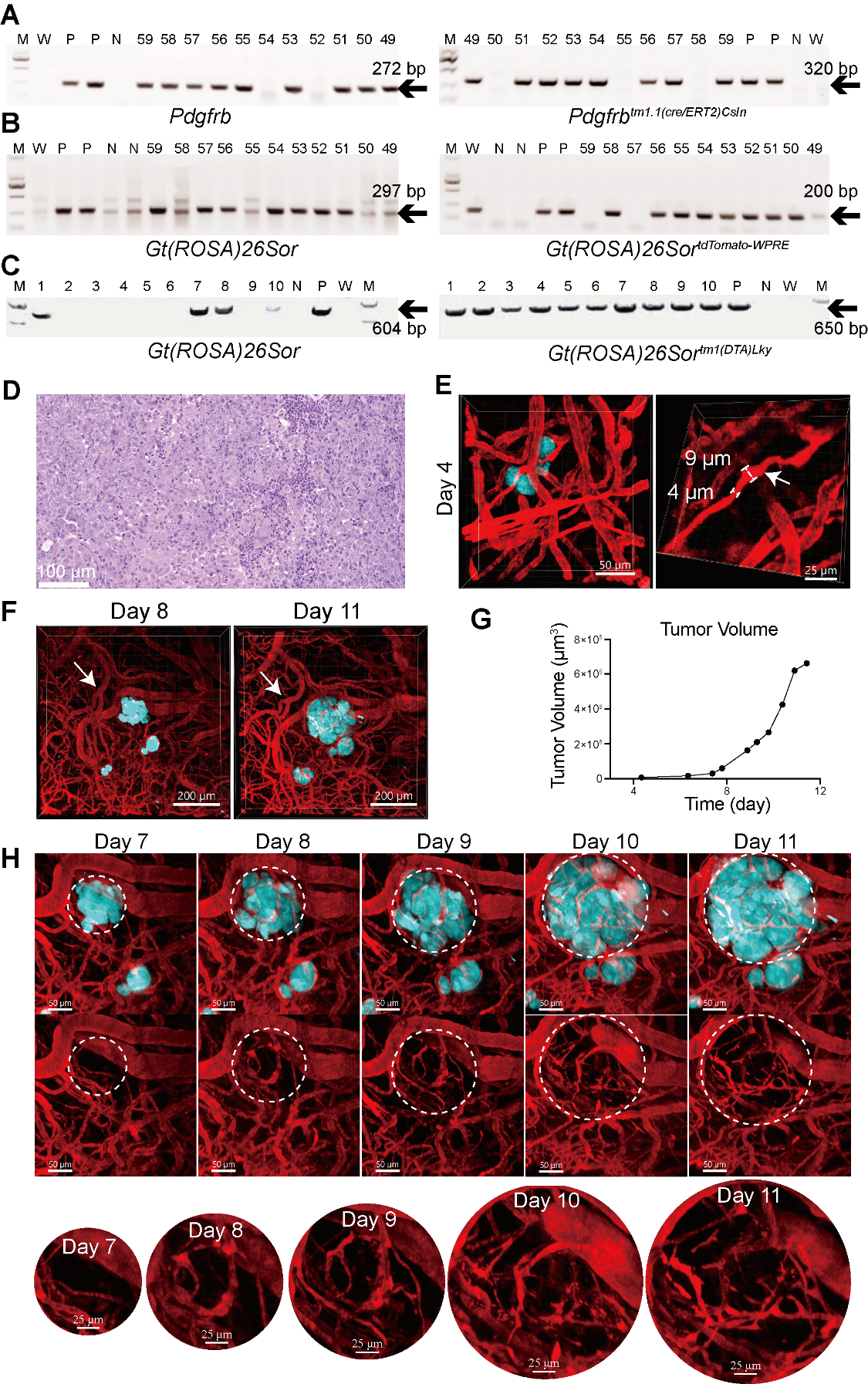
**

(A–C) Genotype identification. (D) H&E-stained GBM and adjacent brain tissue sections. (E) Vascular co-option. (F) Migration of pre-existing blood vessels. (G) Statistical plot of tumor volume changes. (H) Neovascularization.

**Supplementary Figure 2**

**
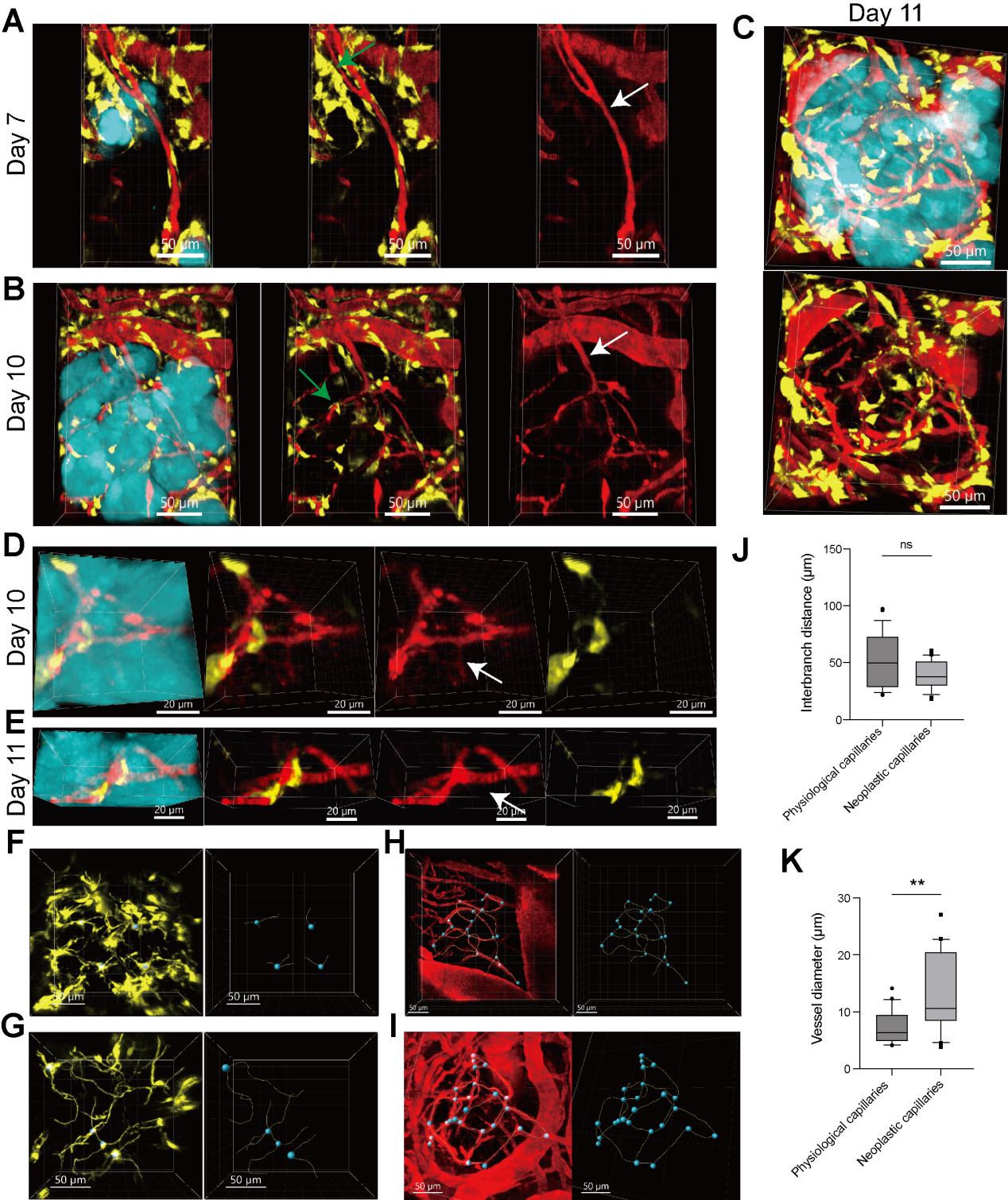
**

Tumor-induced vascular phenotype transition: from single pre-existing blood vessels (A) to tumor capillary networks (B). (C) Representative images of pericyte distribution on tumor capillaries. Process of the same blood vessel from immature (D) to mature (E). Representative 3D reconstruction images of tumor-associated pericytes (F) and brain pericytes (G). Representative 3D reconstruction images of normal capillary networks (H) and tumor capillary networks (I). Statistical plots of blood vessel branch point spacing (J) and blood vessel diameter (K). * p < 0.05, ** p < 0.01, *** p < 0.001.

**Supplementary Figure 3**

**
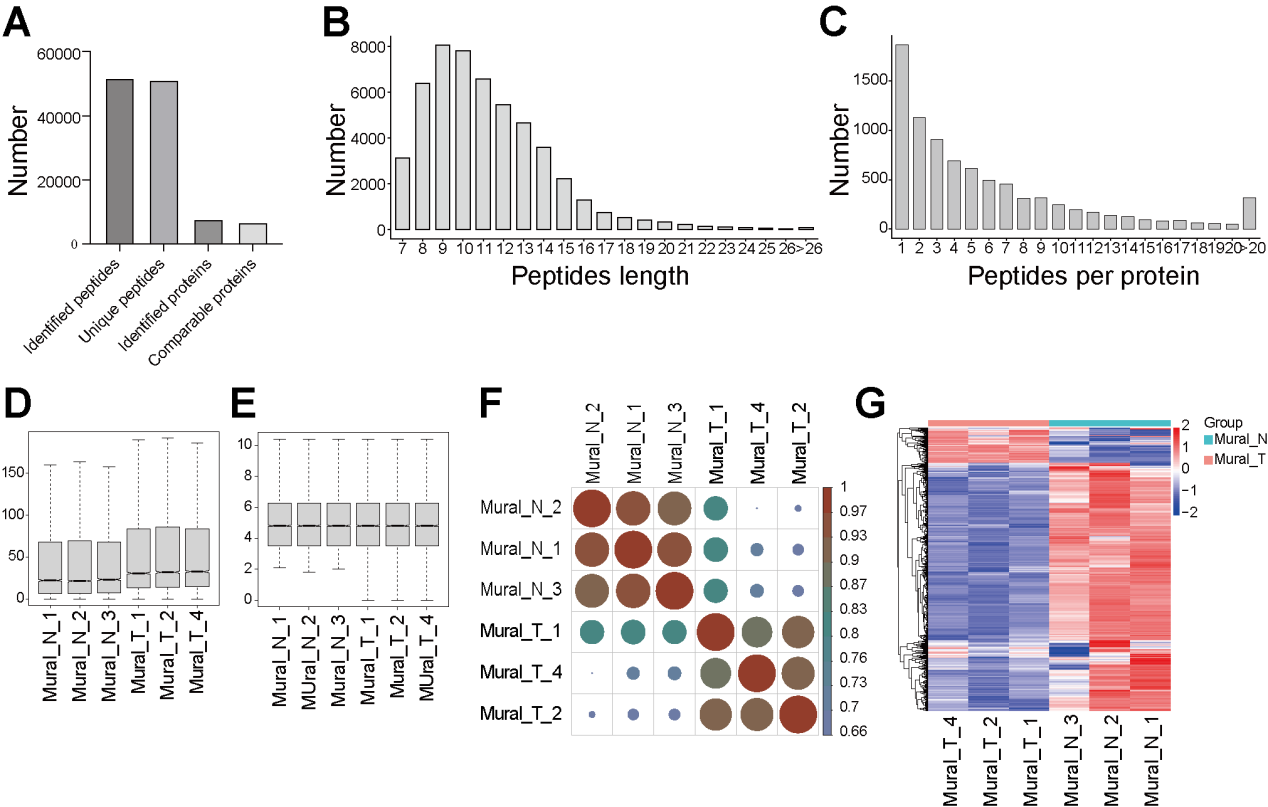
**

Supplementary Figure 3

(A) Shows an overall overview of the number of peptides and proteins identified after data filtering of the database search results. (B) Presents the length distribution characteristics of peptides identified by mass spectrometry. (C) Presents the distribution of the number of peptides corresponding to individual proteins. Box plots of data before (D) and after (E) CPM normalization processing. (F) Heat map of sample correlation. (G) Heat map of sample clustering.
